# Lightweight Porous Glass Composite Materials Based on Capillary Suspensions

**DOI:** 10.3390/ma12040619

**Published:** 2019-02-19

**Authors:** Katharina Hartung, Carolyn Benner, Norbert Willenbacher, Erin Koos

**Affiliations:** 1 Karlsruhe Institute for Technology, Institute for Mechanical Process Engineering and Mechanics, Gotthard-Franz-Straße 3, 76131 Karlsruhe, Germany; katharina.hartung@kit.edu (K.H.); carolyn.benner@yahoo.de (C.B.); norbert.willenbacher@kit.edu (N.W.); 2 Department of Chemical Engineering, KU Leuven, Celestijnenlaan 200f, 3001 Leuven, Belgium

**Keywords:** porous materials, capillary suspensions, glass filters, thermal isolation, microparticles, capillary bridges

## Abstract

In this article, we present a simple, advanced method to produce lightweight tailor-made materials based on capillary suspensions that are made from locally bonded hollow glass spheres with a high total porosity in the range of 70% at apparent densities of 200 kg/m^3^, having a compressive strength of 0.6 MPa. The amount of added liquid and the particle surface treatment determine the network structure in the pastes and the resulting microstructure of the porous material in a straightforward manner. This structure has a strong impact on the porosity, pore size, and mechanical properties of the final body. The most promising porous materials were made of surface treated hollow glass spheres that create a sample-spanning network in the capillary state, where the added liquid wets the particles worse than the bulk fluid. These samples approach the density of natural balsa wood and they may find application in fields where either weight or structure are important, such as in insulation materials, filters, and membranes, as well as lightweight construction materials for automotive or aerospace engineering.

## 1. Introduction

Porous materials are found in a broad field of applications, such as separation and filtering techniques, as well as thermal insulation materials or even catalytic materials [1], while the pore size is a key property. The pore size is categorized in macro- (>50 nm), meso- (50 nm–2 nm), and micro (<2 nm) porous structures [2]. Here, we focus on macroporous materials that are made from glass. Besides the pore size, the pore and inner structure of the material are also important, as they influence the mechanical properties along with the porosity. In the past few years, attention was focused on natural structure architectures, as these extraordinary structured materials evolve properties that are well-suited to specific environmental conditions. Examples include the plate-plate structure of sea shells with a compressive strength of up to 540 MPa [3], hierarchically porous structured bamboo with compressive strength up to 68 MPa at a density of 900 kg/m^3^ [4,5], and highly porous and strong balsa wood with compressive strength of around 40 MPa at densities of 100–250 kg/m^3^ [6,7,8]. Recent approaches have attempted to either directly mimic these natural structures using inorganic materials or to create other complex structures to surpass the material properties of these natural materials. Bauer and coworkers were able to create micron-sized cellular composite materials that are made of three-dimensional (3D) polymer trusses from laser lithography that were coated with alumina [9]. The samples had densities that were below 1000 kg/m^3^ and compressive strengths of up to a 280 MPa. Their micro-architecture, with size of 10 to 40 µm in all directions, is the benefit—and drawback—of these samples, since this method creates stable, but quite delicate, structures. Furthermore, these complex microstructures would be difficult to implement on a rapid, large scale for industrial processes where bodies that are larger than a few centimeters are required. A similar method was also established for pure ceramic materials on a larger scale. Minas et al. were able to produce cellular alumina scaffolds with a hierarchically- structured pore network and high porosity (67–77%) at a compressive strength between 9.5 and 29.3 MPa using three-dimensional (3D) printed Pickering emulsions and foams [10]. While ceramic materials often have superior strength, glass and glass composite materials are preferred in certain applications because of their resistance to chemicals, surface tunability, as well as easy cleaning and reuse. Maurath et al. used the capillary suspension concept, which will be described in the following paragraph, to produce sintered dense glass filters with porosities of up to 50% with a compressive strength of 14.7–21.4 MPa at an average sample density of 1375 kg/m^3^ [11]. Rincón et al. [12] were able to produce porous glass foam materials via alkali activation, gel casting, and sintering at 700–800 °C with porosities of 88–93%, densities from 2110–2660 kg/m^3^, and strengths from 0.5 up to 5 MPa. Qu and coworkers could produce even more efficient lightweight glass foams at equivalent sintering temperatures, but with lower densities of 129–229 kg/m^3^, higher porosities of 91–95%, and compressive strength of 0.85–5.92 MPa [13,14]. While there are advantages in the physical and mechanical properties of these products over traditional bulk manufacturing [15], the high sintering temperatures present clear drawbacks. Therefore, a simpler and more energy efficient processing route should be explored. 

As mentioned above, further improvements on bulk forming methods are possible while using the capillary suspension processing route. Capillary suspensions are created by adding a small amount of an immiscible secondary fluid to a particulate suspension, thereby modifying rheological properties of the suspension [16,17]. The changes can be detected by, e.g., a strong increase of the yield stress, which is caused by a transition from fluid-like to gel-like behavior due to structure formation within the suspension. This small amount of secondary liquid phase is distributed as capillary bridges between the micron-sized particles, leading to a particle network due to the capillary attraction. This percolating network of particles and capillary bridges was directly visualized using the confocal microscopy by Bossler and Koos [18]. They show the two extrema in the shape of the capillary bridges between the particles in the suspension, depending on the three-phase contact angle, where the secondary phase forms between the particles. The first case is the so-called capillary state for a three-phase-contact angle $\theta_{S,B}>90{}^{\circ}$, where the secondary fluid (S) wets the particle surface worse than the surrounding bulk fluid (B). If the secondary fluid preferentially wets the particles ($\theta_{S,B}<90{}^{\circ}$), then the particles are directly connected by capillary bridges and the system is in the pendular state. Both of the arrangements lead to a sample-spanning network, but they show significant differences in the overall structure. The pendular state is characterized by individual capillary bridges between the particles (binary interactions), while the capillary state often appears in multibody particle clusters [17,19,20,21]. Capillary suspensions in the pendular state have already been used to produce porous materials by either sintering or locally fusing the microparticles [22]. A proof of concept for direct polymerization within the capillary bridges was shown by Hauf et al. [23], where a simple method for direct polymerization in the bridges at temperatures below 100 °C, requiring less time and effort than existing methods, is presented. Hauf et al. investigated the chemistry, including the chemical composition and molecular weight distribution of poly(methyl methacrylate) (PMMA) capillary bridges between glass particles to demonstrate how the bridge structure and the resulting porous body properties could be tuned. Bitsch et al. were successful in using a reactive epoxy resin as the secondary fluid to connect plate shaped graphite particles, resulting in lightweight conductive open porous bodies (ε=60 –75%), with a compressive strength of 0.1–1 MPa [22]. 

In this paper, we present an advanced method using capillary suspensions to create self-organized porous bodies with open porosities of up to 67% at an apparent density of only 200 kg/m^3^ and a compressive strength of 0.6 MPa. These samples are made of micron-sized hollow glass spheres that are locally connected by two-component epoxy bridges. The density of our glass-epoxy materials approach that of balsa wood and it is comparable with common insulation materials, like foamed poly(styrene), but they have superior resistance against chemicals, solvents, or thermal exposure, in addition to the easy surface modification. Furthermore, the process route is very simple, low cost, consumes little energy, and is environmentally friendly. 

## 2. Experimental Section

### 2.1. Materials

Hollow, borosilicate glass spheres iM16k (untreated) and iM16k OTES (treated with octyltriethoxysilane) were provided by 3M Deutschland GmbH (Neuss, Germany) with a mean diameter $d_{50}=20$ μm and a density of 0.46 g/cm^3^, differing in surface treatment. Particles were used as received and are shown in the supplementary information Figures S1 and S2. As shown in the supporting information, the spheres have a rough surface consisting of small glass particles that were adsorbed onto the micro-particle surface. This roughness appears to be more pronounced for the treated particles than the untreated, but these nanoparticles can migrate during processing [23]. Glycerol (Carl Roth, Karlsruhe, Germany), with a density of 1.26 g/cm^3^, was used as the bulk phase and a two-component epoxy as the secondary phase. The epoxy base (Epoxydharz L) and curing agent (Härter L) were provided by Conrad Electronic SE (Hirschau, Germany). The interfacial tension between epoxy and glycerol was measured to be Γ = 8 mN/m. 

### 2.2. Sample Preparation

To prepare the capillary suspensions, the particles were dispersed in glycerol with a high-shear dissolver stirrer (35 mm diameter for rheological measurements and 60 mm diameter for the cured samples) at 1200 rpm for 10 min, with a solid volume fraction of 30 vol.%. The two-component epoxy was premixed at a ratio of 10:4 by weight with a spatula and then added to the suspension while further mixing at 2000 rpm for 2 min. The amount of secondary fluid $\phi_{\sec}$ was 0, 3, 4.5, 6, 7.5, or 9 vol.%. 

The prepared capillary suspensions were filled into silicone molds (35 mm diameter disks with 5 mm height for porosity measurements, bars of 48 mm × 12 mm × 5 mm for the mechanical experiments) and cured (crosslinking of the epoxy) in the mold for two days at ambient conditions. The glycerol was then removed from the cured samples by placing them on paper towels for at least three hours and then rinsing the samples twice with distilled water. Finally, they were dried overnight in a laboratory oven at 70 °C and 200 mbar. 

### 2.3. Characterization

Yield stress measurements were performed with the stress controlled rheometer Haake RS 150 (Thermo Scientific, Karlsruhe, Germany) using a vane geometry (10 mm diameter) by increasing the stepwise shear stress in a range from 0.1 to 100 Pa for approximately 20 min. The temperature for all measurements was T = 20 ± 0.5 °C. The yield stress was defined using the tangential method [22]. The values reported are for at least two unique measurements.

The three-phase contact angle between the glycerol and epoxy (without hardener) was determined by imaging particles at the interface between the two fluids using a microchannel and confocal microscope (Leica TCS SP8, Leica Microsystems, Wetzlar, Germany) [18]. The contact angle was determined to be 19 ± 4° for the untreated glass hollow spheres and 110 ± 10° for the treated particles. The interfacial tension between glycerol and the epoxy was determined using the pendent drop method with image analysis from Krüss (Krüss, Drop Shape Analysis, Hamburg, Germany).

The samples were also optically inspected after curing using an environmental scanning electron microscope (ESEM-mode at p=70 Pa, Quanta 650 FEG, FEI, Hillsboro, OR, USA), where the images were taken from a fractured section of the solid samples. The samples were sputtered with a mixture of platinum and palladium prior to imaging.

The total porosity ε of the solid bodies was calculated using the ratio of the apparent (raw) density of the sample and the true (skeletal) density based on DIN EN 1936.

(1) $$\varepsilon_{\mathrm{total}}=1-\;\frac{\rho_{\mathrm{apparent}}}{\rho_{\mathrm{true}}}$$

Here, the apparent density of the sample was calculated as the ratio of sample weight and sample volume. The true density was measured using a multivolume gas pycnometer (Micromeritics GmbH, Aachen, Germany, Model MP 1305 with Helium) at 20 °C. Pore size measurements were carried out using the mercury porosimeter Autopore III 9420 (Micromeritics GmbH, Aachen, Germany) by detecting the intrusion volume at a mercury filling pressure of 3.9 kPa. 

The compressive strength was measured using the texture analyzer TAX.T from Stable Micro Systems (Surrey, UK) in compression mode with an average sample size of 4 mm height, 7 mm length, and 7 mm depth for at least five samples at a constant laboratory temperature of 20 ± 1 °C. The flexural strength was obtained from four-point bending tests using a custom-made device following DIN EN 843-1 that was mounted on the TAX.T. 

## 3. Results and Discussion

**Figure 1:**
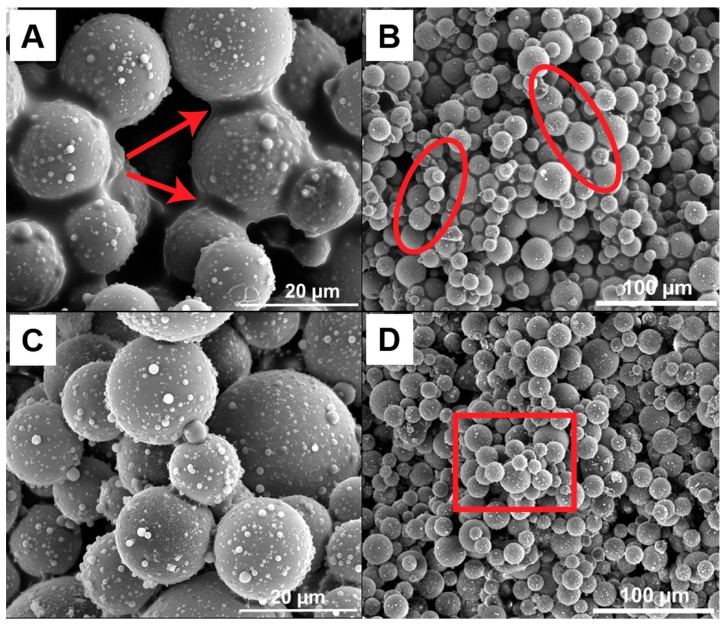
Images of fractured sections of the cured porous glass materials made from 30 vol.% untreated hollow glass spheres and 3% (**A**,**B**) and 6% (**C**,**D**) epoxy. The samples were cured at ambient conditions for at least 24 h and the bulk phase was removed prior to imaging.

**Figure 2:**
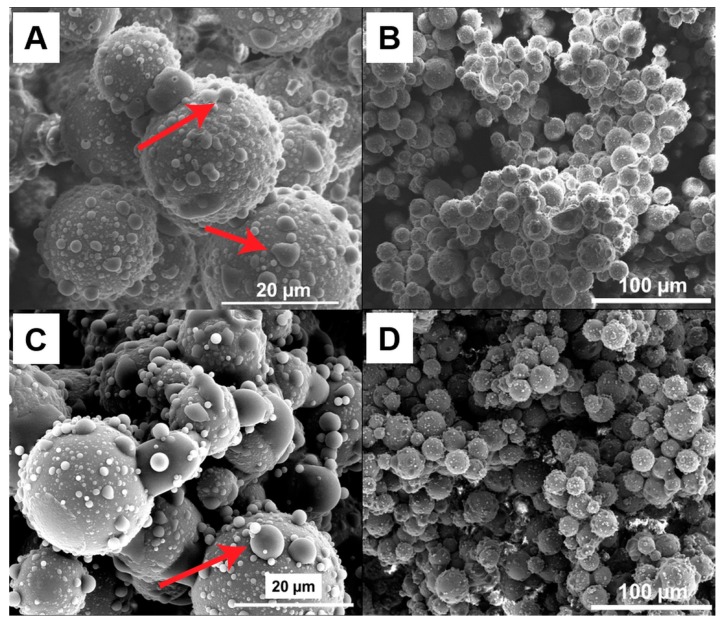
Images of fractured sections of the cured porous glass materials made with 30 vol.% treated and 3% (**A**,**B**) and 6% (**C**,**D**) epoxy. The samples were cured at ambient conditions for at least 24 h and the bulk phase was removed prior to imaging.

Lightweight, strong, and highly porous glass bodies can be produced using hollow glass micro spheres that are locally interconnected with nano-sized epoxy bridges. The porous materials were made from capillary suspensions containing glass spheres, glycerol as bulk fluid, and an epoxy resin as secondary phase, inducing the formation of a strong sample-spanning network. High energy dispersing of a small amount of epoxy in this suspension $\left(\phi_{\sec}/\phi_{\mathrm{solid}}=0.1\;\mathrm{to}\;0.3\right)$ leads to a break-up of the epoxy droplets and to the attachment of the resulting small droplets between the particles [24]. With the different surface treatment of the hollow glass spheres, the microstructure of the particulate network can be controlled due to the different wetting behavior of the epoxy on the particle surface. This effect is clear when comparing the SEM images displaying θ<90° for the untreated particles in Figure 1 and θ>90° for the treated particles in Figure 2. The contact angle of the cured samples is consistent with the three-phase contact angles (19 ± 4° and 110 ± 10°) that were measured in the liquids. The true contact angle, however, may differ because of the contact angle hysteresis (advancing vs. receding polar phase) as well as the contact line pinning that may occur during the mixing process [25]. The SEM images suggest a different bridging mechanism between the untreated and treated particles as well as a difference in the structure morphology as the amount of secondary liquid is varied. For the untreated particles that are shown in Figure 1, the epoxy creates concave capillary bridges (highlighted with arrows) between the untreated particles (Figure 1A) and a funicular network (highlighted with circles) is built with 3 vol.% epoxy (Figure 1B).

The funicular network, as described in Domenech and Velankar [26], and subsequently imaged in Bossler and Koos [18], consists of secondary fluid drops with low sphericity connecting several particles. The epoxy wets the particles very well, showing a low three-phase contact angle, which would facilitate the formation of a funicular network at low $\phi_{\sec}/\phi_{\mathrm{solid}}$ [18].

At higher amounts in the secondary phase, here, 6%, the epoxy seems to partially flood the particle network forming agglomerates. Only small amounts of epoxy are visible in forming direct bridges between particles in the high magnification image (Figure 1C). Instead, a majority of the epoxy is on the inside of the large agglomerates (highlighted with squares), which are visible in Figure 1D. Domenech and Velankar observed a similar phenomenon while using silica particles to produce liquid-liquid-particle ternary blends containing two immiscible polymers [27]. They found several distinct morphologies upon varying the composition [27], and this image, Figure 1D, seems to resemble the capillary aggregates that they described. 

A different wetting behavior was observed on the surface of the treated particles in Figure 2. The epoxy connects multiple particles forming large, irregular agglomerates. The epoxy is also seen in Figure 2A forming sessile drops (highlighted with arrows) on the particle surface and connections between the larger micron-sized particles and the smaller, nanometer-sized particles on the particle surface. With an increasing amount of secondary liquid to 6% epoxy, the number and size of the sessile drops changes, but the agglomerate structure does not seem to vary significantly.

**Figure 3:**
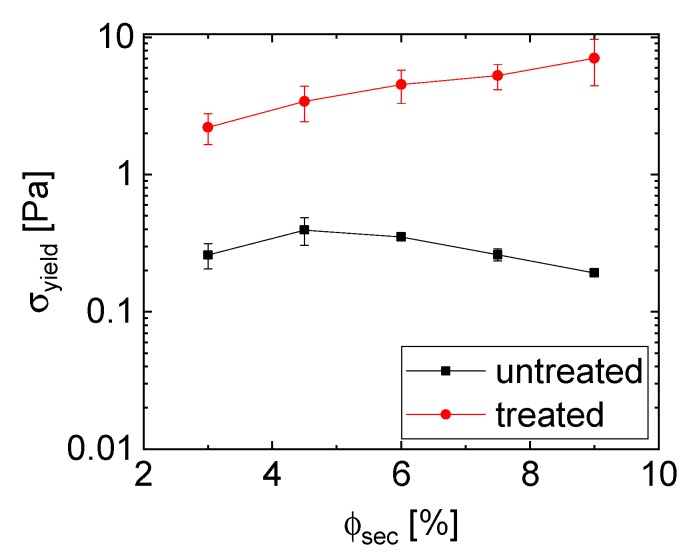
Yield stress of capillary suspensions made from untreated (black squares) and treated (red circles) glass spheres ($\phi_{solid}=30$ vol.%) in glycerol with the epoxy base (without curing agent) as secondary phase. The value for the suspensions below 3 vol.% epoxy could not be measured indicating a lack of structure formation. Lines are to guide the eye.

The yield stress is closely related to the samples’ microstructure [28]. The changes in structure following a variation of the amount of epoxy are manifested in measurements of the yield stress of the wet, uncured samples [28]. Thus, the network structure and bridge strength are indirectly detected by rheological measurements showing increasing yield stress with increasing amounts of epoxy, as seen in Figure 3. 

The measured yield stress values are relatively low in comparison to other capillary suspensions [11,18], which is likely because of the low interfacial tension (Γ=8 mN/m) between epoxy and glycerol or the influence of the nanoparticles on the particle surface interfering with the wetting and bridging of the microparticles. The qualitative comparison between capillary suspensions with untreated and treated glass spheres shows that the particulate network using treated hollow glass spheres in glycerol with epoxy as secondary phase creates a stronger network than with untreated particles. This trend is the opposite to that demonstrated by Bossler and Koos [18], where the pendular state samples had a higher magnitude of the shear modulus than the capillary state samples. Subsequent experiments imply that this difference might be highly sensitive to the amount of added liquid; however, and the capillary state networks can indeed be stronger than the pendular state samples [28].

The rheological behavior of the capillary suspensions with treated glass spheres shows a slight, continuous increase in yield stress with an increasing amount of secondary phase, while the yield stress of the pastes with untreated particles shows a minor peak at 4.5% epoxy ($\phi_{\sec}/\phi_{\mathrm{solid}}=0.15)$ and then a decrease in strength. This is typical for a pendular to funicular transition—the transition from binary bridging interactions to coalesced bridges with multibody interactions—in pendular state (wetting) capillary suspensions, and typically occurs at $\phi_{\sec}/\phi_{\mathrm{solid}}=0.1\;\mathrm{to}\;0.25$, depending on the three phase contact angle and the mixing conditions [18,24,29]. The SEM images that are shown in Figure 1A and Figure 2B suggest that funicular clusters are already present at 3% epoxy $(\phi_{\sec}/\phi_{\mathrm{solid}}=0.1$), implying that this may not be a pendular to funicular transition. Instead, we hypothesize that this transition denotes a microstructural transition to an agglomerated, capillary aggregate structure with either fewer or weaker bridging between the particle clusters. The steady increase in yield stress for the treated sample implies that no clear microstructural transition occurs in this range for the capillary state sample, and instead, either the strength or the number of bridging interactions steadily increases. SEM images in Figure 2 showing no clear change in structure between 3% and 6% epoxy confirm such an observation.

The difference in σ_yield_ between the two different particle wetting types may be explained by the different network structures that are created by the secondary phase. Capillary state structures were observed on the treated particles (Figure 2). They are visible as convex drops between the particles and small droplets sitting on the particles surface. These drops between the particles (termed capillary state clusters, which are not to be confused with the capillary clusters for the untreated particles) also lead to the formation of a sample-spanning network [18], as shown in Figure 2B. With increased secondary liquid ($\phi_{\sec}=6\%)$, the drops on the particle surface become larger and more numerous (Figure 2C). These drops may reinforce the connections between the particles. 

**Figure 4:**
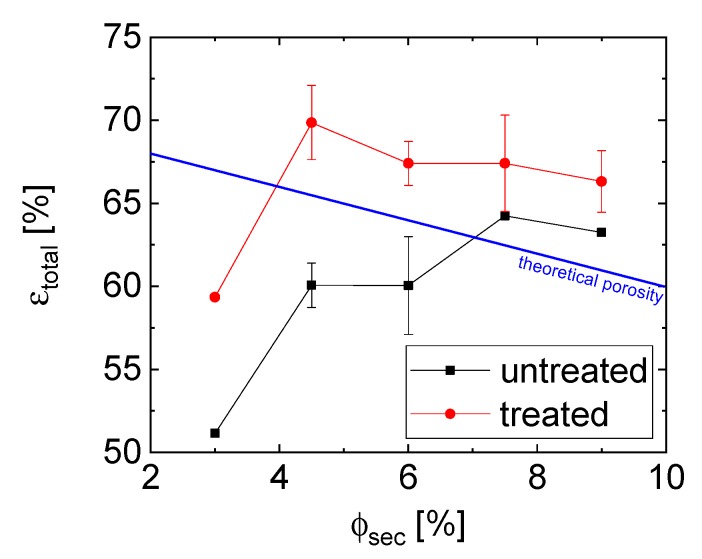
Influence of the amount of epoxy on the open porosity of the glass materials made of untreated particles (black squares) and treated (red circles). The values for the 0 vol.% were not measured because there no self-supporting bodies could be produced. Lines are to guide the eye. The theoretical maximum open porosity line (blue) denotes the maximum porosity that is the volume of the bulk fluid, assuming there is no phase separation or shrinkage.

The morphological changes also influence the porosity and the pore size distribution of the cured sample. The total porosity of the glass materials with treated particles was higher, by up to 10%, than that of the bodies that were made of untreated ones (Figure 4). The porosity drastically increases between 3 vol.% to 4.5 vol.% of added sec. fluid, from 59 to 69% for the treated particles and from 51 to 60% for the untreated particles. Further increasing the bridging phase for the treated samples does not significantly influence the porosity, but it may change the pore structure, as shown by the higher standard deviation between samples (error bars). The samples with the treated particles also have a higher volume of air bubbles that were entrapped during sample preparation, which may account for the higher porosity when compared to the samples that were made with untreated particles. For the untreated samples, the peak porosity occurred at a higher epoxy amount, which can be explained by the changing network structure that was caused by spherical agglomeration or by partial phase separation effects. The low yield stress of the precursor with untreated particles could be an indication of either possibility. A line (calculated by $100\%-\phi_{\mathrm{solid}}-\phi_{\mathrm{epoxy}}$), denoting the maximum theoretical open porosity if the glycerol (bulk fluid) is removed without phase separation or shrinkage, is also included in Figure 4. Due to the structural properties of capillary suspensions, we assume a predominantly open pore structure [30]. The points at low secondary fluid below this line comprise evidence for phase separation due to the weak yield. The contribution to the closed porosity from the hollow glass spheres is estimated to be 26.5% (as calculated using SEM images of broken particles showing a shell thickness of 0.4 μm) and is constant for all samples. This could be the reason for porosity values above the theoretical open porosity line. 

**Figure 5:**
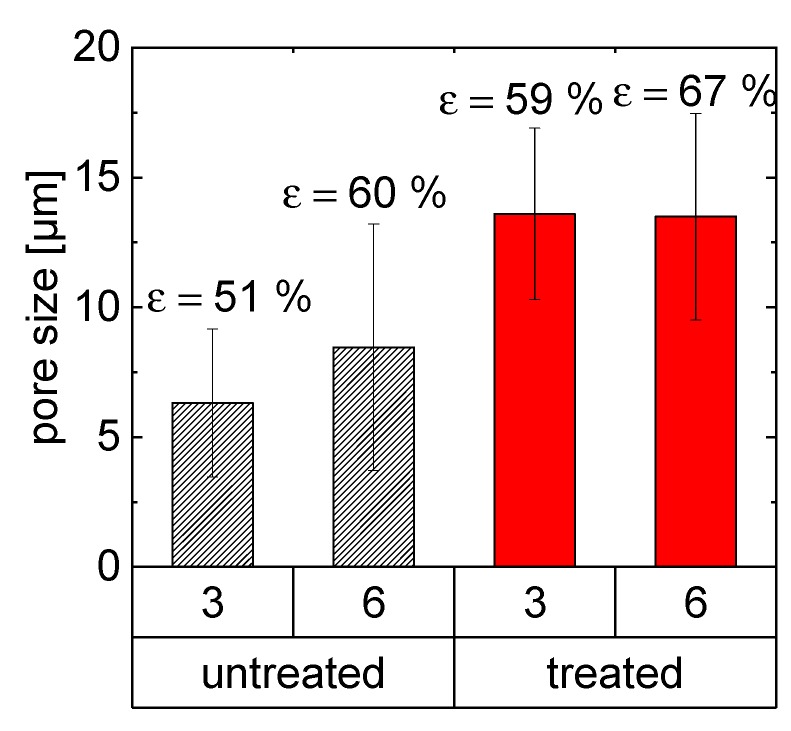
Pore size measurements of untreated and treated samples with 3 and 6 vol.% epoxy. The pore size distributions were monomodal, with the exception of 6% untreated, which had a bimodal distribution with peaks at 8 µm and 90 µm (see supplementary information). The error bars denote the standard deviation as calculated from the pore size distribution.

Structural differences between the samples become clear from average pore size measurements, as shown in Figure 5, where error bars represent the standard deviation of the pore size distribution. The 3% untreated sample has the lowest median pore size of 6 µm at a porosity of 51%, while the 6% sample has an average pore size of 8 µm and a porosity of 60%. This slight increase in the average pore size is caused both by the slight increase in the size of the small sized pores with size 8 µm as well as the development of a bimodal distribution with large pores of 90 µm. The bimodal distribution (as shown in the supplementary information in Figures S3 and S4) indicates agglomeration and the formation of the capillary aggregates, as was demonstrated for other systems [16,28]. The treated particles, on the other hand, show a similar porosity of 59%, like the untreated with 6% epoxy, but a larger pore size of 14 µm at 3% epoxy (supplementary information Figures S5 and S6). The average pore size stays approximately constant and the porosity increases up to 67% for the 6% treated sample with an increasing amount of epoxy. 

The differences between the untreated and treated samples in porosity and pore size distribution can be explained by the difference in the density of the bodies. The average apparent and the true densities of the treated and untreated samples at all secondary fluid fractions are shown in Table 1. Here, higher densities for the samples from the untreated particles as compared with the treated particles can be observed. The apparent density is 15% and the true density 33% higher for the untreated particles. Therefore, the untreated samples are more compact than the treated ones, corresponding to a lower porosity in the solid bodies. 

**Figure 6:**
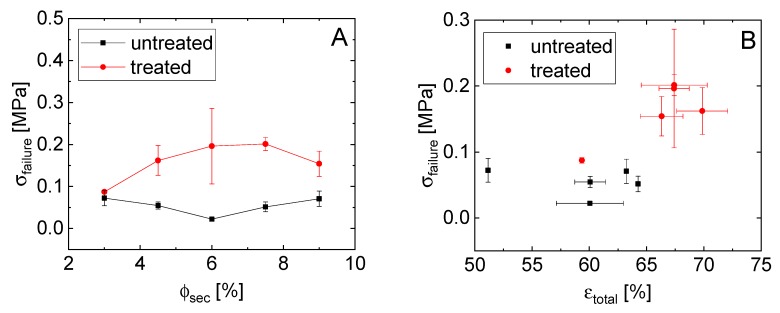
(**A**) Dependence of the four-point-bending failure strength on the secondary phase amount and (**B**) the correlation between the failure strength and the open porosity of the solid glass materials.

The addition of the epoxy to the glass particles induces a sample-spanning network and, after curing, results in a self-supporting body. Changes in the pore structure as well as the size and number of bridges with changing amounts of epoxy should manifest in changes to the material strength, as is observed when using measurements of the flexural and compressive failure strength. Under flexural stress, the sample will crack, where this crack propagates through the weakest particle connections. Measurements of the four-point-bending failure strength are shown in Figure 6A as a function of $\phi_{\sec}$. The treated particle bodies show a much higher resistance against bending, with a maximum of 0.2 MPa for 7.5 vol.%, while untreated materials had their maximum of 0.07 MPa with only 3% epoxy. 

The failure stress, which is a measure of the strength of the network backbone, is higher for the treated particle samples at each $\phi_{\sec}$ than for the untreated particles (Figure 6A). Remarkably, the treated particle samples show both higher porosity and strength (Figure 6B). While a capillary network is created through the use of treated particles with a small amount of epoxy, these bridges seem to become thicker and the contact area increases with higher secondary fluid fractions, thus causing increased adhesion between the particles. The small epoxy droplets that are shown in Figure 2 become larger with an increasing amount of epoxy and create a stronger cluster-network, which incorporates more epoxy volume [28]. Both the treated and untreated particle samples can be divided into two populations when comparing their failure strength to porosity (Figure 6B). The higher $\phi_{\sec}$ samples form one group at higher porosity while the sample with the lowest epoxy fraction (3 vol.%) clearly deviates. Interestingly, both the strength and porosity increase for the higher epoxy contents as compared to the $\phi_{\sec}=3\%$ sample for the treated particles. This clearly demonstrates the importance of the network backbone on the strength.

However, it is worth noting that, besides the trend where the treated samples were stronger than the untreated, the dependence of the bending failure has little in common with the yield stress dependence. The treated sample bending failure has a peak or plateau beginning at 7.5 vol.%, whereas the yield stress steadily increases over the measured range. For the untreated particles, the yield stress increases slightly, from 3% to 4.5% epoxy, and then weakens, while the failure strength is constantly weak during the entire range. These differences may arise due to the contribution of flexible bridges, which can break and reform in the wet state, or simply from the difference in the geometry of the applied force in these measurements.

**Figure 7:**
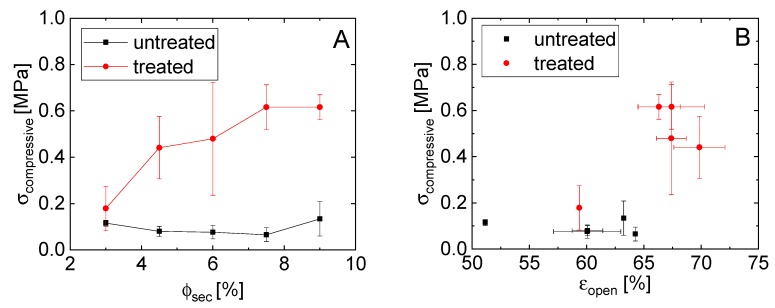
(**A**) Dependence of compressive strength on the secondary phase amount and (**B**) correlation between the compressive strength and the open porosity of the solid glass materials.

A similar trend was observed for the compressive strength of the porous glass bodies, as shown in Figure 7. While the compressive strength for the untreated materials remains essentially in constant in the range from 0.1 to 0.04 MPa with increasing epoxy, the compressive strength of the treated samples increases from 0.18 to 0.62 MPa (Figure 7A). Once again, this strength is related to the structure and volume of the capillary bridges. The compressive failure decreases slightly with an increased porosity for the untreated samples, whereas the 3% epoxy treated sample had the lowest porosity and strength (Figure 4 and Figure 7B).

**Figure 8:**
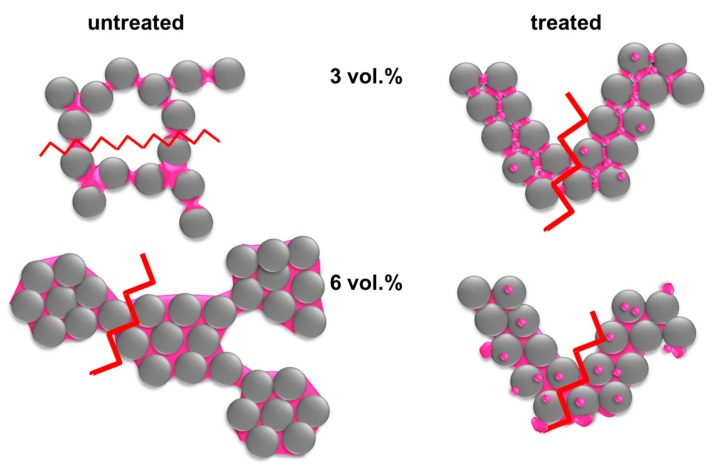
Schematic drawing of structure arrangement and corresponding failure mechanism in capillary suspensions with untreated and treated particles, at low and high secondary fluid contents. The zig-zag-lines show the location of mechanical failure.

Thus, using the data from both the cured and uncured samples, we may construct the microstructural schemes, as shown in Figure 8. The untreated particles are wet by the epoxy secondary phase, which creates pendular and funicular bridges between the spheres. The largest contact area for adhesion occurs between 3–4.5% epoxy (highest $\sigma_{\mathrm{yield}}$). The structure at this point is either a pendular or funicular structure, where each secondary fluid bridge connects a small number of particles. This structure changes upon the addition of more epoxy into a highly aggregated state. The epoxy wets the untreated particles very well and individual bridges will already start to coalesce at small $\phi_{\sec}/\phi_{\mathrm{solid}}$. These coalesced bridges will cover the particles, forming compact clusters [18]. This compact clustering causes the change in porosity and a bimodal pore size distribution was observed for the 6% sample (see Figure 5 and the supporting information). Some binary bridges remain in this sample that connect the clusters [18]. These are the binary connections that will fracture. Thus, there is a large change in porosity between the funicular and spherical aggregate sample, Figure 6B, while the strength is only weakly affected. For the treated particles, the strength and number of the bridges increases slightly with increasing epoxy, but the general structure, as evidenced by the porosity and the pore size, remains unchanged.

**Figure 9:**
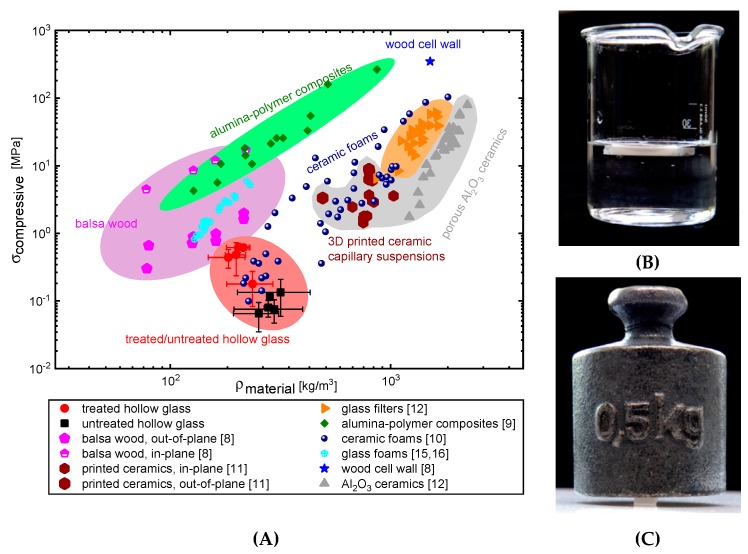
(**A**) Compressive strength as a function of density of the glass-epoxy samples, honeycomb-structured sintered ceramics [32] and polymers [9], sintered glass foams [13,14] and Balsa wood [8]. The produced treated glass-epoxy-materials are much lighter compared with sintered ceramics. (**B**,**C**) Images of samples, demonstrating their low density and high strength. (**B**) The image on top right shows a 50 mm × 20 mm × 5 mm bar floating in distilled water and (**C**) the right bottom image show a 48 mm × 12 mm × 5 mm bar supporting a 0.5 kg weight.

The treated glass spheres create a capillary network upon the addition of small amounts of epoxy, which enables cured products that are highly porous, very lightweight, and nevertheless strong against bending and compression, as shown in Figure 9. The most promising material was made of 30 vol.% treated hollow glass spheres (d_50_ = 20 µm) with 7.5 vol.% epoxy $\left(\phi_{\sec}/\phi_{\mathrm{solid}}=0.25\right)$. At an apparent density of only 200 kg/m^3^, it had a failure strength of 0.2 MPa and a compressive resistance of 0.6 MPa at a porosity of 67.4%. The glass-epoxy materials favorably compare with the density of lightweight balsa wood [6,7,8]. This low density and reasonable strength is remarkable, having a porous material that is made of locally bonded hollow glass spheres without any sintering step. While the sintered glass materials showed stronger mechanical properties [11], the locally glued glass-epoxy-materials are 6.9 times lighter, with almost 18% higher porosity, with controllable pore shapes and without any shrinkage problems. Commercial glass frits (e.g., DURAN Group GmbH (Mainz, Germany), Heraeus Quarzglas GmbH & Co. KG (Kleinostheim, Germany)) were measured to have a porosity of 29% and a compressive strength of around 20 MPa, which is even less than for the sintered glass filters from capillary suspensions [11]. Hemmerle et al. [31], who used 55–2040 µm sized glass beads ($\rho_{p}=2460–2560$ kg/m^3^) with PDMS as the bridging phase, have shown similar values of 0.025–0.35 MPa compressive strength, but at much higher densities than the hollow glass samples that are shown here. When compared with the glass foams from Rincón et al. [12] and Qu and co-workers [13,14], our porous samples are in the same range of weight and mechanical properties and they have the advantage of a simple and environmentally friendly processing route. 

By soaking these bodies in ethanol, acetone and n-hexane for more than 30 h, the chemical resistance was tested. The thermal resistance was tested by heating up the samples in an evacuated laboratory oven at a temperature of 70 °C for 24 h. No fracturing or other degradation of the samples was observed during either treatment. This resistance depends on the chemical composition of the bridging material (epoxy in this case), and it can be modified to provide stability against thermal and chemical agents. 

## 4. Conclusions

Our glass-epoxy-system demonstrates an advanced way to produce glass filters and other porous glass composite materials with tailor-made properties. The glass hollow spheres render the material lightweight, while also retaining the desired surface properties, allowing for capillary suspension networks to be formed and creating the desired pore structure. In this study, a commercial anaerobic two component epoxy was used. The processing route using capillary suspensions gives a variety of variables to control the pore network, the porosity, the mechanical properties, and even the chemical resistance of the resulting porous bodies. The low density and a reasonable compressive strength are in good agreement with the most lightweight materials, with the exception of state-of-the-art materials, like the high-sintering glass foams or 3D printed composite structures using more tedious lithographic printing methods. Our processing route, however, is much simpler and it can be easily transitioned to large-scale industrial processes. Possible applications for this porous material include separation and filtration, as well as lightweight construction for automotive and aerospace engineering, or insulation materials. These materials are made of hollow glass spheres that have a similar thermal conductivity of 0.05–0.25 W/mK, which is in the same range as foamed polystyrene (0.20–0.38 W/mK). The glass bodies are resistant to chemicals, solvents, or thermal exposure. Furthermore, the process route is very simple, low cost, consumes little energy, and is environmentally friendly.

Further research should be completed to improve the strength and further reduce the density. A possible approach to improve the strength can be to increase the adhesion between the glass spheres and the bridging phase by choosing a different epoxy or by modifying the particle treatment. The density could be lowered by 3D printed structured materials. The application of specific experiments should also be conducted, especially at different temperature and humidity ranges, where the strength of the glass and epoxy might vary. The strength of this material under tension should also be examined. 

## Figures and Tables

**Table 1:** Average apparent and true densities as well as the resulting porosity of porous samples made with untreated and treated particles as a function of the epoxy fraction $\phi_{\sec}$. The data are the average values from at least three samples.

Surface Treatment	$\Phi_{\sec}\;[\%]$	$\rho_{\mathrm{apparent}}\;[g/{\mathrm{cm}}^{3}]$	$\rho_{\mathrm{true}}\;[g/{\mathrm{cm}}^{3}]$	$\varepsilon_{\mathrm{total}}\;[\%]$
untreated	3.0	0.28 ± 0.00	0.72 ± 0.12	51.1 ± 0.00
	4.5	0.28 ± 0.01	0.70 ± 0.01	60.1 ± 1.34
	6.0	0.30 ± 0.10	0.65 ± 0.09	60.0 ± 2.94
	7.5	0.25 ± 0.06	0.65 ± 0.08	64.2 ± 0.07
	9.0	0.32 ± 0.12	0.71 ± 0.10	63.2 ± 0.00
treated	3.0	0.24 ± 0.06	0.62 ± 0.07	59.3 ± 0.09
	4.5	0.17 ± 0.02	0.57 ± 0.02	69.9 ± 2.18
	6.0	0.20 ± 0.02	0.59 ± 0.03	66.6 ± 3.35
	7.5	0.20 ± 0.02	0.62 ± 0.02	67.4 ± 2.89
	9.0	0.21 ± 0.02	0.64 ± 0.01	67.3 ± 1.16

## Supplementary Materials

The following are available online at http://www.mdpi.com/1996-1944/12/4/619/s1, Figure S1: SEM image of the untreated particles; Figure S2: SEM image of the treated particles; Figure S3: Pore size distribution of the untreated particles with 3% epoxy; Figure S4: Pore size distribution of the untreated particles with 6% epoxy; Figure S5: Pore size distribution of the treated particles with 3% epoxy; Figure S6: Pore size distribution of the treated particles with 6% epoxy.
