# Supplementary material for: Lightweight Porous Glass Composite Materials Based on Capillary Suspensions
Source: Materials (Basel). 2019 Feb 19;12(4):619. doi: 10.3390/ma12040619 (PMC6416639; doi:10.3390/ma12040619)
Supplement: Supplementary file 1 [file materials-12-00619-s001.pdf]

Lightweight Porous Glass Composite Materials based on Capillary Suspensions

Katharina Hartung [1], Carolyn Benner [1], Norbert Willenbacher [1] and Erin Koos [2,*]

[1] Karlsruhe Institute for Technology, Institute for Mechanical Process Engineering and Mechanics, Gotthard-Franz-Straße 3, 76131 Karlsruhe, Germany; katharina.hartung@kit.edu (K.H.); carolyn.benner@yahoo.de (C.B.); norbert.willenbacher@kit.edu (N.W.)
[2] Department of Chemical Engineering, KU Leuven, Celestijnenlaan 200f, 3001 Leuven, Belgium.
* Correspondence: erin.koos@kuleuven.be

Supporting Information

- SEM images of original particles
- Pore size distributions

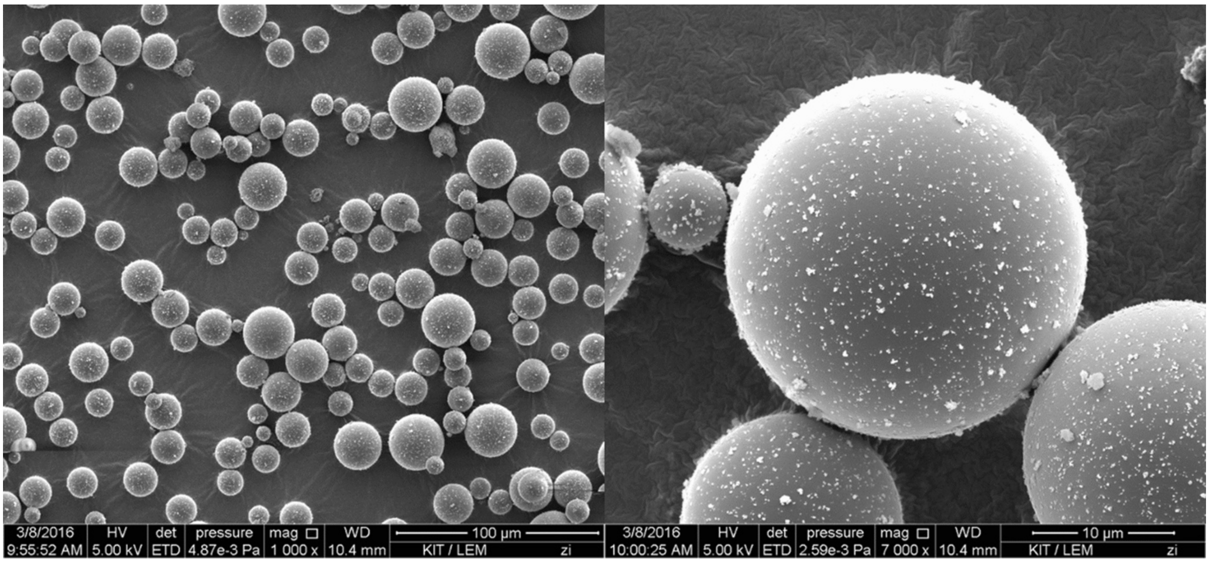

Figure S1. SEM image of the untreated particles

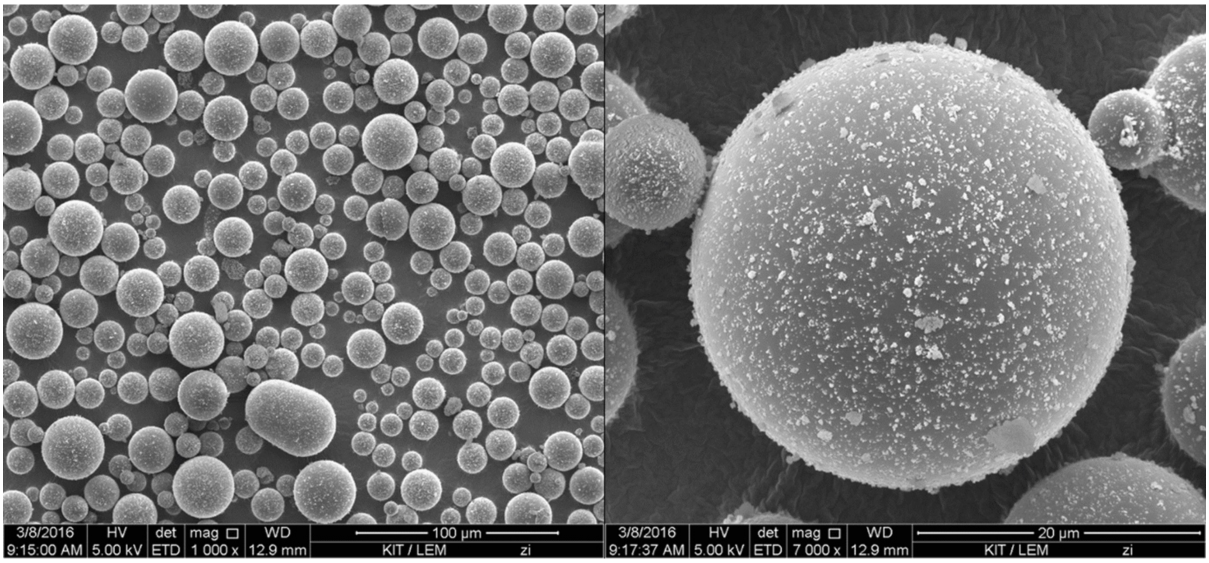

Figure S2. SEM image of the treated particles

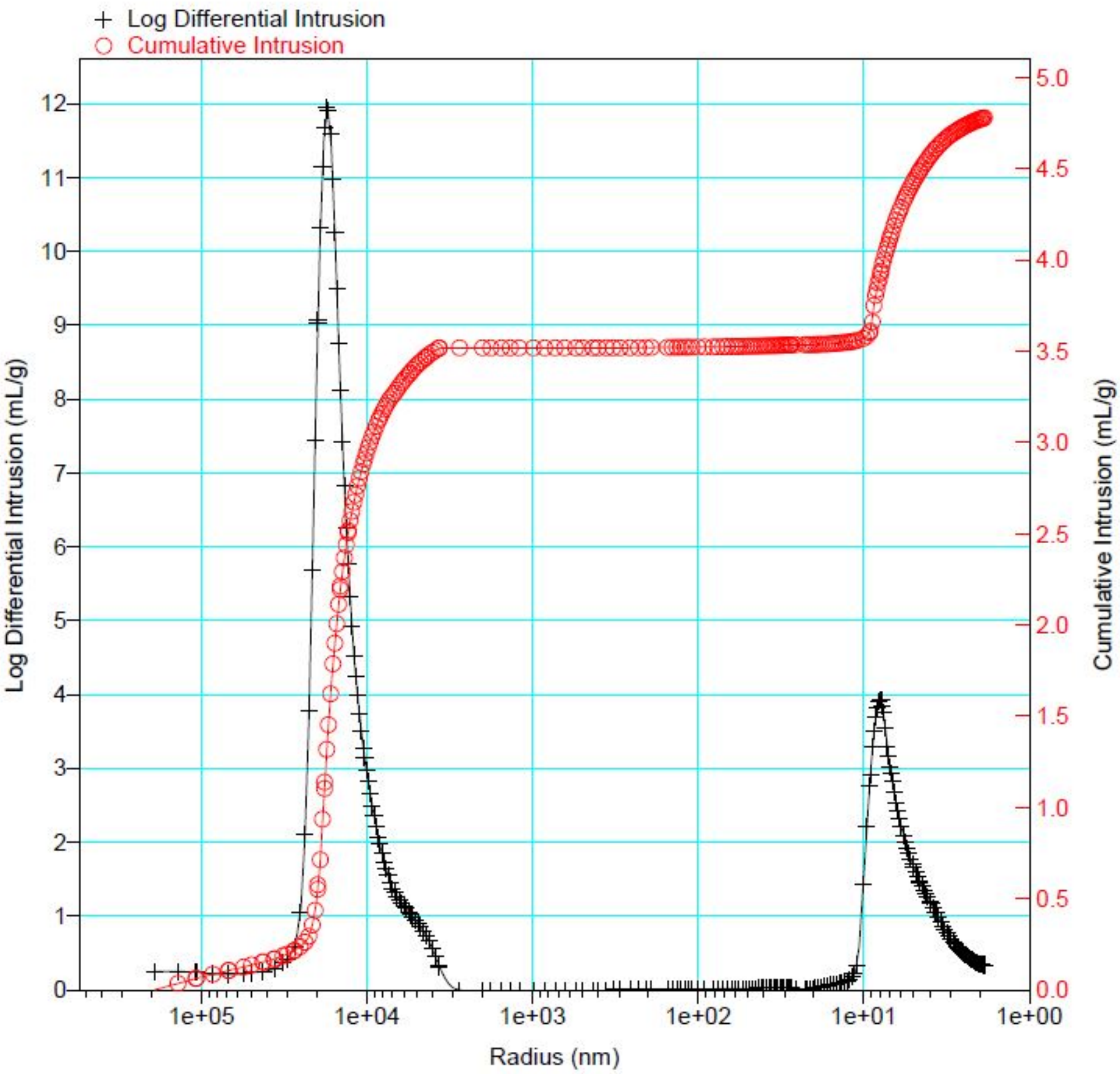

Figure S3. Pore size distribution of the untreated particles with 3% epoxy

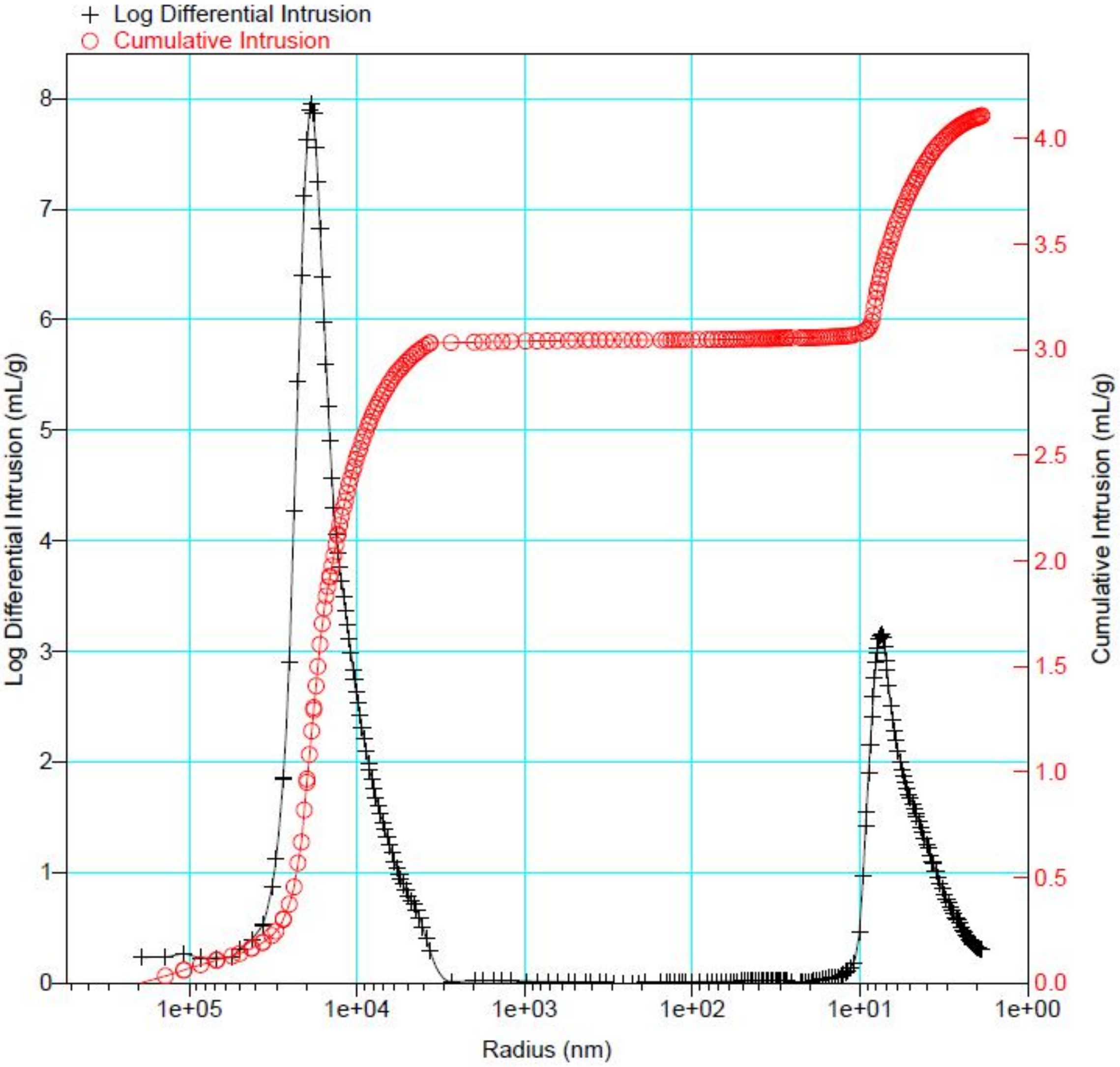

Figure S4. Pore size distribution of the untreated particles with 6% epoxy

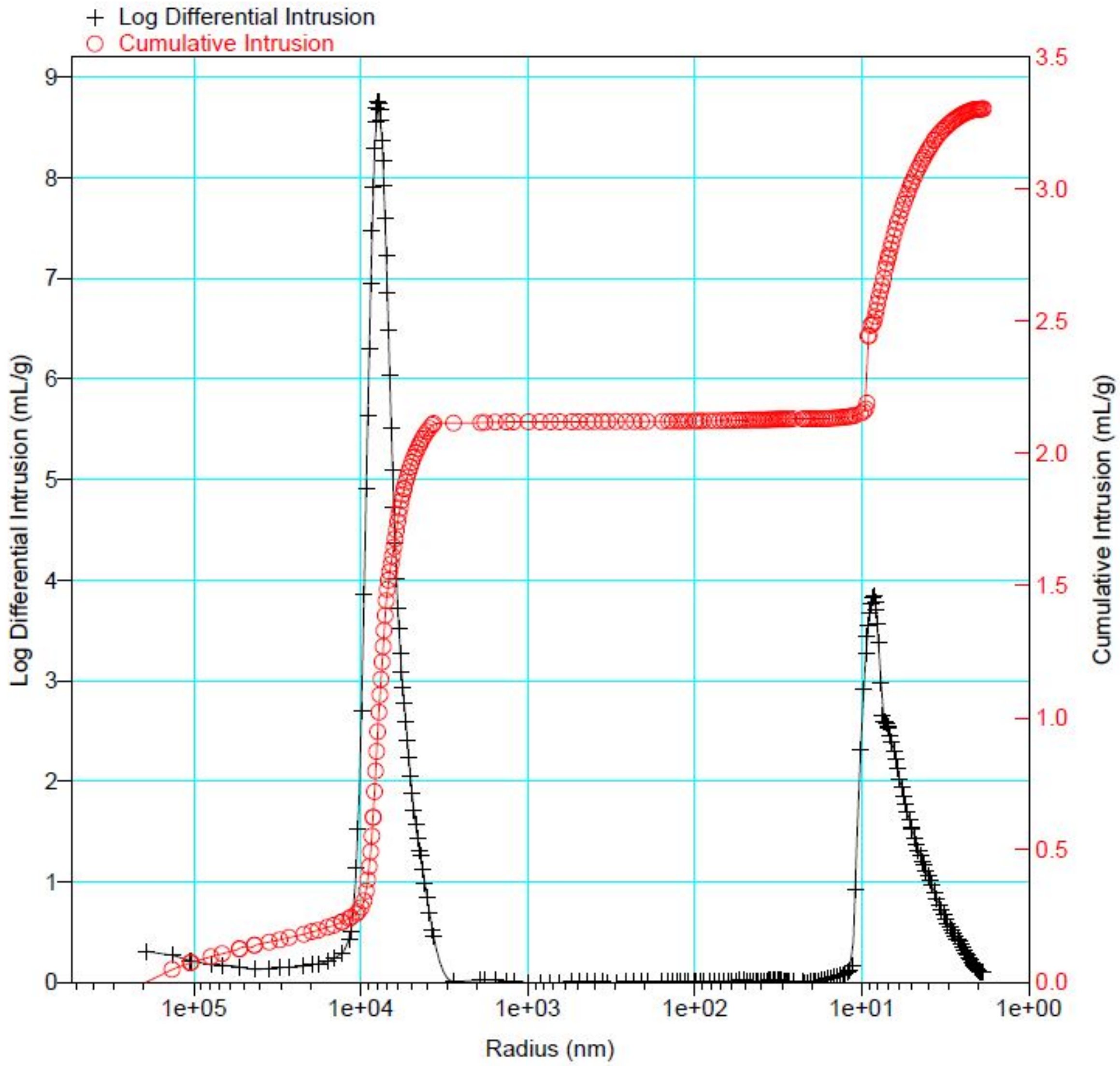

Figure S5. Pore size distribution of the treated particles with 3% epoxy

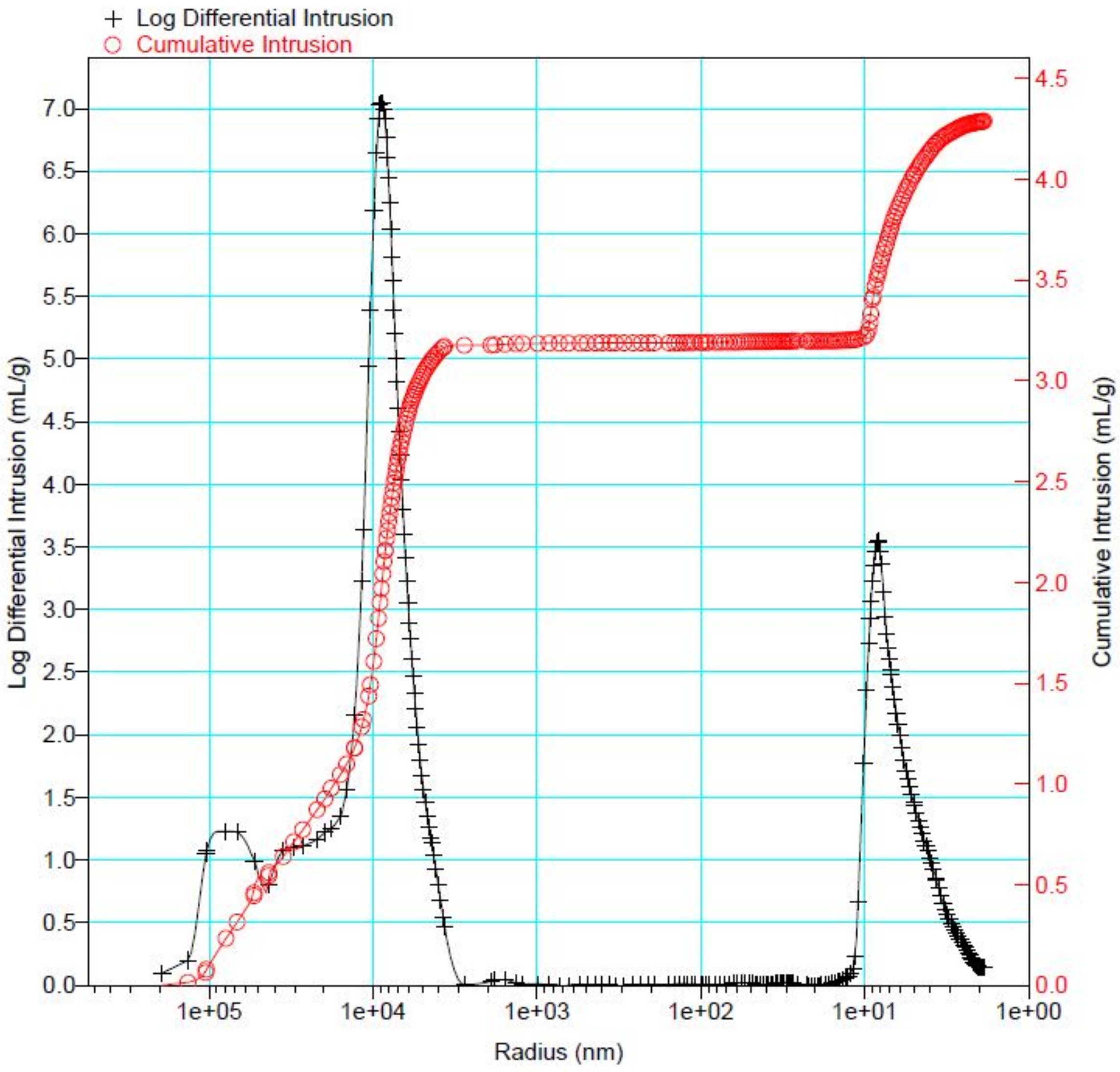

Figure S6. Pore size distribution of the treated particles with 6% epoxy
